# Supplementary material for: Global hypo-methylation in a proportion of glioblastoma enriched for an astrocytic signature is associated with increased invasion and altered immune landscape
Source: eLife. 2022 Nov 22;11:e77335. doi: 10.7554/eLife.77335 (PMC9681209; doi:10.7554/eLife.77335)
Supplement: Figure 2—source data 1. [file elife-77335-fig2-data1.zip › Figure_2_source_data_1/Figure_2A/homerResults/motif13.similar.html]

motif13

## Information for motif13

C
A
G
T
T
A
C
G
A
T
G
C
T
C
G
A
A
C
T
G
A
G
C
T
T
A
G
C
C
A
G
T
C
A
T
G
C
A
T
G
A
T
C
G
G
T
A
C
G
T
C
A
C
A
T
G
T
A
C
G
A
T
G
C
A
T
G
C
A
T
G
C
C
G
T
A
A
T
C
G
A
T
C
G
T
A
G
C
T
A
G
C
A
T
G
C
G
C
A
T
T
A
C
G
G
C
A
T
A
T
C
G
T
A
G
C
G
T
A
C
G
A
T
C
C
T
A
G
T
A
C
G
T
A
G
C
T
C
A
G
T
A
C
G
C
A
G
T
A
T
C
G
T
C
G
A
T
C
A
G
T
A
C
G
A
T
G
C
G
T
C
A
T
A
G
C
A
G
C
T
  
Reverse Opposite:  

T
C
G
A
A
T
C
G
C
A
G
T
T
A
C
G
A
T
G
C
A
G
T
C
A
G
C
T
T
A
G
C
G
T
C
A
A
T
G
C
A
G
T
C
A
T
C
G
A
T
G
C
A
G
T
C
C
T
A
G
C
A
T
G
A
T
C
G
T
A
G
C
C
G
T
A
A
T
G
C
C
G
T
A
A
T
C
G
A
T
C
G
A
T
C
G
A
T
G
C
T
A
G
C
G
C
A
T
T
A
C
G
T
A
C
G
T
A
C
G
A
G
T
C
G
T
A
C
C
A
G
T
C
A
T
G
T
A
G
C
G
T
A
C
G
T
A
C
G
T
C
A
A
T
C
G
T
C
G
A
T
G
A
C
A
G
C
T
T
A
C
G
A
T
G
C
G
T
C
A
  

|  |  |
| --- | --- |
| p-value: | 1e-22 |
| log p-value: | -5.210e+01 |
| Information Content per bp: | 1.476 |
| Number of Target Sequences with motif | 20.0 |
| Percentage of Target Sequences with motif | 0.86% |
| Number of Background Sequences with motif | 1.5 |
| Percentage of Background Sequences with motif | 0.04% |
| Average Position of motif in Targets | 90.5 +/- 46.3bp |
| Average Position of motif in Background | 163.0 +/- 0.0bp |
| Strand Bias (log2 ratio + to - strand density) | 0.0 |
| Multiplicity (# of sites on avg that occur together) | 1.20 |
| Motif File: | file (matrix) reverse opposite |

### Similar de novo motifs found

|  |  |  |  |  |  |  |  |
| --- | --- | --- | --- | --- | --- | --- | --- |
| Rank | Match Score | Redundant Motif | P-value | log P-value | % of Targets | % of Background | Motif file |
| 1 | 0.754 | C A G T T C A G T A C G C T A G C T A G A T G C G T A C C A G T A T C G A T C G A C T G A G T C G C T A A C T G T A C G A T C G C A T G A G C T A G C T A T C G T A G C A T G C G T A C A T G C G C A T T A C G A C T G A C T G T A G C G A T C T A G C C G T A T C A G A T G C T G C A T A C G C G T A T A C G T C G A A C T G | 1e-20 | -47.418509 | 0.99% | 0.08% | motif file (matrix) |
| 2 | 0.704 | A C G T A T C G A T G C C A T G A T C G A G C T G T A C C A G T A T C G C T A G C A T G A T G C G C T A C A T G A C T G C A G T A C G T A C G T A C G T T A C G A T G C G T A C G T C A A G T C C A G T A T C G A G C T T A C G A T G C A G T C G A T C C G T A C A T G T A C G T G C A A T C G C A G T T A C G C T G A A C T G A T G C A T C G G C A T A G T C C A G T C A G T T A G C G A C T A T C G G A T C | 1e-19 | -45.229039 | 0.78% | 0.00% | motif file (matrix) |
